# Is Surgical Treatment for Obesity Able to Cure Urinary Incontinence in Women?—A Prospective Single-Center Study

**DOI:** 10.3390/life13091897

**Published:** 2023-09-11

**Authors:** Cristian Persu, Remus Nicolae Cartas, Irina Ciofu, Bogdan Mastalier, Victor Mihail Cauni

**Affiliations:** 1 Department of Urology, “Carol Davila” University of Medicine and Pharmacy, 020021 Bucharest, Romania; cristian.persu@umfcd.ro; 2 Department of Urology, Colentina Clincal Hospital, 020125 Bucharest, Romaniadrcauni@gmail.com (V.M.C.)

**Keywords:** urinary incontinence, bariatric surgery, obesity, stress incontinence, urgency incontinence, mixed incontinence

## Abstract

There is enough evidence to support weight loss in order to improve urinary incontinence. Nevertheless, weight loss and maintaining a lower weight are not easy to achieve in the general population. Our study aims to evaluate whether bariatric surgery has a positive effect on the symptoms of urinary incontinence in female patients. We performed a prospective study on obese female patients before and after bariatric surgery, over a period of 9 years. Patients with a BMI ≥ 33 kg/m^2^ were included if they described involuntary loss of urine and no previous surgery for urinary incontinence was performed. The patients underwent laparoscopic surgery, either gastric sleeve, bypass or banding, performed by four surgeons in our hospital. The type of incontinence was not assessed at the initial visit carried out by the surgeon. All patients who declared being incontinent were referred to the urologist where they received the ICIQ—UI-SF questionnaire before their bariatric surgery and during follow -up visits. The sum of points obtained at questions 3, 4 and 5 was used to evaluate the severity of incontinence, as well as the impact on the quality of life. Our evaluation collected data on age, time since onset of symptoms, pad usage, number and type of deliveries, concomitant conditions and medications. The type of incontinence was assessed by the urologist before bariatric surgery as urge, stress or mixed incontinence. At follow-up visits, the patients were also asked to fill out a 10-point VAS questionnaire evaluating their perception on the evolution of incontinence symptoms. Data were analyzed using *t*-test statistical analysis. Our objective defined changes in incontinence as cure, improved, no change and worse. We included 54 women from whom initial data and at least 18 months of follow-up were available. We observed that about 50% of all women undergoing bariatric surgery have some degree of urinary incontinence. The ICIQ score improved from 13.31 ± 5.18 before surgery to 8.30 ± 4.49 points after surgery (*p* < 0.0001). Before surgery, 38 patients (70%) described severe incontinence compared to only 20 patients (37%) after surgery. A total of 16 women (31%) reported complete cure of urinary incontinence after bariatric surgery. Data from the VAS questionnaire show improvement in 46 cases (85%). Pad usage improved from 7.04 ± 2.79 to 3.42 ± 2.77 (*p* < 0.001) per day. The number of patients using more than one pad per day decreased from 35 (65%) to 9 (17%). The type of incontinence did not seem to be relevant, but our sample size was too small to lead to statistically significant results. There was no impact on the outcome of incontinence of number/type of delivery, age or BMI. Our data show that bariatric surgery is able to cure urinary incontinence in one of three obese women. A significant improvement was obtained in more than two-thirds of the patients, regardless of the type of incontinence. For an obese female with urinary incontinence, treatment for obesity should prevail and incontinence should be treated only if symptoms remain.

## 1. Introduction

Urinary incontinence (UI) is defined as an involuntary loss of urine, representing a social or hygienic concern. There are three main types of UI: stress urinary incontinence (SUI), urgency urinary incontinence (UUI) and mixed urinary incontinence (MUI). SUI represents involuntary urination in response to effort, physical activity, sneezing or coughing, whilst UUI is defined as the involuntary leakage of urine preceded or accompanied by an intense desire to urinate [1,2]. According to the literature reports, about 50% of adult women could suffer from a form of UI at some point in their life [3]. Due to the nature of the female urinary tract and risk factors like pregnancy, childbirth and hysterectomy that can harm the connective tissue and musculature of the pelvic floor, the prevalence of UI in women is almost double than in men [4].

Obesity is an important health issue with an increasing prevalence worldwide [5,6]. It is defined as excess fat accumulation that raises the likelihood of adverse health outcomes. Classification of obesity uses body mass index (BMI, determined by dividing the body weight in kilograms by height in meters squared). A BMI ≥ 30 kg/m^2^ defines obesity, while a BMI ≥ 40 kg/m^2^ indicates severe obesity [7]. According to current trends, obesity will afflict 51.1% of people in the USA by 2030, up from more than one-third of adults today [8]. It decreases life expectancy and predisposes people to a number of diseases [9]. Obesity is an independent risk factor for all types of urinary incontinence [10,11]. Incontinence has been found to affect as many as 60% to 70% of severely obese women [12]. With every five-point increase in body mass index (BMI), the odds of UI rise by 30–60% [13].

Depending on the type of UI, there are different treatment options in obese patients. Surgery is still the basis for treating SUI, whether it be through the insertion of a midurethral sling, Burch colposuspension, pubovaginal sling using autologous rectus fascia or the use of a bulking agent. However, women with a BMI over 35 kg/m^2^ have a relatively high failure rate for this procedure: 81% of them manage to gain and maintain continence, compared to 92% to 96% of the general population [1,14,15]. Pharmacotherapy, primarily with anticholinergic drugs, is the main form of treatment for UUI. Treatment for MUI is still challenging and often involves a combination of surgery and medication, depending on the primary complaint [1]. Weight loss, bladder training and pelvic floor muscle development are all known to help patients regardless of the type of UI [16]. Weight-loss therapies have been observed to lessen the frequency of UI in obese women [17,18,19]. The strongly recommended course of treatment for women with UI with a BMI over 30 kg/m^2^ is lifestyle management, according to professional organizations such the European Association of Urology (EAU) and the National Institute for Health and Care Excellence (NICE). This includes suggestions for weight loss through nutritional, pharmaceutical and behavioral treatment, or a mix of these interventions [20,21]. However, only about 20% of persons who adhere to lifestyle changes are able to maintain long-term weight loss of at least 10% [22].

For patients with morbid obesity (BMI ≥ 40 kg/m^2^), as well for individuals with a BMI ≥ 35 kg/m^2^ and related co-morbidities, bariatric surgery is the most effective method for long-term weight loss [23,24]. Interventions to lose weight have been demonstrated to lessen the frequency of UI [17,18,19]. In a randomized controlled clinical trial, Subak et al. concluded that the treatment for obese women with UI that involves weight loss is successful. Weight loss of 5% to 10% should be thought of as a first-line therapy for incontinence because it has an efficacy comparable to other nonsurgical treatments [17]. In a similar study, Wing et al. concluded that through a weight-loss program, stress incontinence episodes were less frequent over the course of 12 months, and patient satisfaction with incontinence modifications was increased over the course of 18 months [18]. We acknowledge the benefits of losing weight through lifestyle changes, such as a healthier diet with a low-calorie intake or physical exercises for UI. However, patient compliance with these recommendations is very low. As easy as it is to recommend losing weight, it is as difficult to take the steps needed to actually achieve it.

Given the fact that it is proven that weight loss is beneficial for improving symptoms of UI [17,18,19], we intend to assess if bariatric surgery could lead to similar benefits. Our study aims to prospectively evaluate whether bariatric surgery has a positive effect on the symptoms of urinary incontinence in obese female patients.

## 2. Materials and Methods

We conducted a prospective study on obese female patients with symptoms of urinary incontinence, comparing data before and after bariatric surgery over a period of 9 years. Bariatric surgery was carried out in compliance with our hospital standard of care. Written informed consent was signed by all participants and ethical committee approval was obtained prior to the start of the study.

Patients qualified for bariatric surgery by meeting the following requirements: BMI value of 33 kg/m^2^ or above; age between 18 and 55 years old; motivation to have surgery; potential of life-long follow-up; adequate cognitive ability to comprehend the procedure and its implications; and absence of drug or alcohol addictions. The eligible BMI value for surgery was set by our bariatric surgeons. We included in our study only female patients that simultaneously met the bariatric surgery criteria and described involuntary loss of urine at least two or three times a week, as per the ICIQ, and had no previous history of surgery for urinary incontinence.

Patients presented at the hospital with their weight-related issues. Four bariatric surgeons from our clinic made the initial assessment of the patients. A comprehensive medical history and physical examination of the patient were part of the evaluation methodology, including a basic (“Yes”/“No”), non-leading question, on whether they suffer from involuntary loss of urine. Then, a series of laboratory tests were carried out, including a urinalysis, urine culture, complete blood count, serum biochemistry and coagulation test. Patients with pre-diagnosed conditions (e.g., diabetes, high blood pressure) or conditions diagnosed during the pre-operative evaluation were referred to a multi-disciplinary examination before surgery. All participants who described involuntary loss of urine during the surgical assessment were referred to the urology department and assessed by the same urologist. The bariatric surgery was carried out using the laparoscopic approach. Gastric sleeve, bypass or banding were the preferred methods. The procedure technique was chosen by the surgeon, in accordance with our standard of care and the patient’s desire.

During the urological evaluation, we assessed comprehensive medical history regarding UI symptoms. A full clinical examination was performed for each patient. For the purpose of our study, it was decided upfront to exclude patients with pelvic organ prolapse, fistulas or other malformations of the urinary tract. Patients were also required to fill in the International Consultation on Incontinence Questionnaire-Urinary Incontinence short form (ICIQ-UI SF) before and after surgery. A validated Romanian version of the questionnaire was used. The ICIQ-UI SF is a questionnaire that distinguishes between different types of incontinence based on patient self-reporting and assesses the burden of the symptoms on the patient [25]. Female patients that described urine leakage at least two or three times a week (Question nr. 3 from ICIQ-UI SF) were included in our study. Medical history, physical examination and the ICIQ-UI SF were used prior to bariatric surgery to diagnose the type of UI: stress, urge, or mixed incontinence. For the purpose of our study, it was decided upfront to exclude patients with fistulas or other malformations of the urinary tract. The sum of points obtained at questions 3, 4 and 5 was used to evaluate the severity of incontinence, as well as the impact on quality of life. Question nr. 3 assesses the frequency of urine leakage (“Never”—0 to “All the time”—5). Question nr. 4 estimates the amount of urine leaked in patient’s perception (“None”—0 to “A large amount”—6). Question 5 rates how much leaking urine interferes with the patient’s everyday life (“Not at all”—0 to “A great deal”—10). These three separate scores added together result in an overall score between 0 and 21. A lower score indicates a better outcome for symptom severity: mild (1–5), moderate (6–12), severe (13–18) and very severe (19–21) [26]. A similar postoperative ICIQ-UI SF questionnaire was used at follow-up visits. At the first follow-up assessment (1 month after the surgery), we also asked patients to evaluate their perception of urinary symptoms after the surgery. Patients were asked to fill a 10-point visual analogue scale (VAS) regarding how their urinary symptoms evolved (1—It is worse; 5—Same as before; 10—No incontinence). The VAS questionnaire was applied during every follow-up visit after the surgery. For our analysis, we gathered data on age, rural/urban area, height, weight, BMI, gynecologic and obstetric history (including the number and method of births), concomitant conditions and medications, time since onset of incontinence symptoms and pad usage. It is important to underline that no treatment for UI was initiated at this time as that would influence the final outcome of our study. Patients with ongoing treatment for UI were recommended to maintain the same dosage and method of administration. If at the initial evaluation urinary tract infections or other conditions of the lower urinary tract were identified, they were treated, and evaluation was restarted from the beginning, using the same algorithm. Invasive urodynamics, imaging or endoscopy of the urinary tract were not considered as being necessary since no actual treatment for incontinence was planned at this point.

Our primary objective was to evaluate whether bariatric surgery has a positive effect on the symptoms of urinary incontinence. In order to achieve this, we analyzed the results before and after the surgery using the ICIQ-UI SF score, the number of used pads/day and the prevalence of severe incontinence. *t*-test statistical analysis was used in order to compare the collected data before and after the surgery. We used the standard *p* value of 0.05 to determine the degree of statistical significance for each test. For continuous data, the mean and standard deviation were provided, and for categorical variables, the frequency and percentage were reported.

## 3. Results

**Figure 1:**
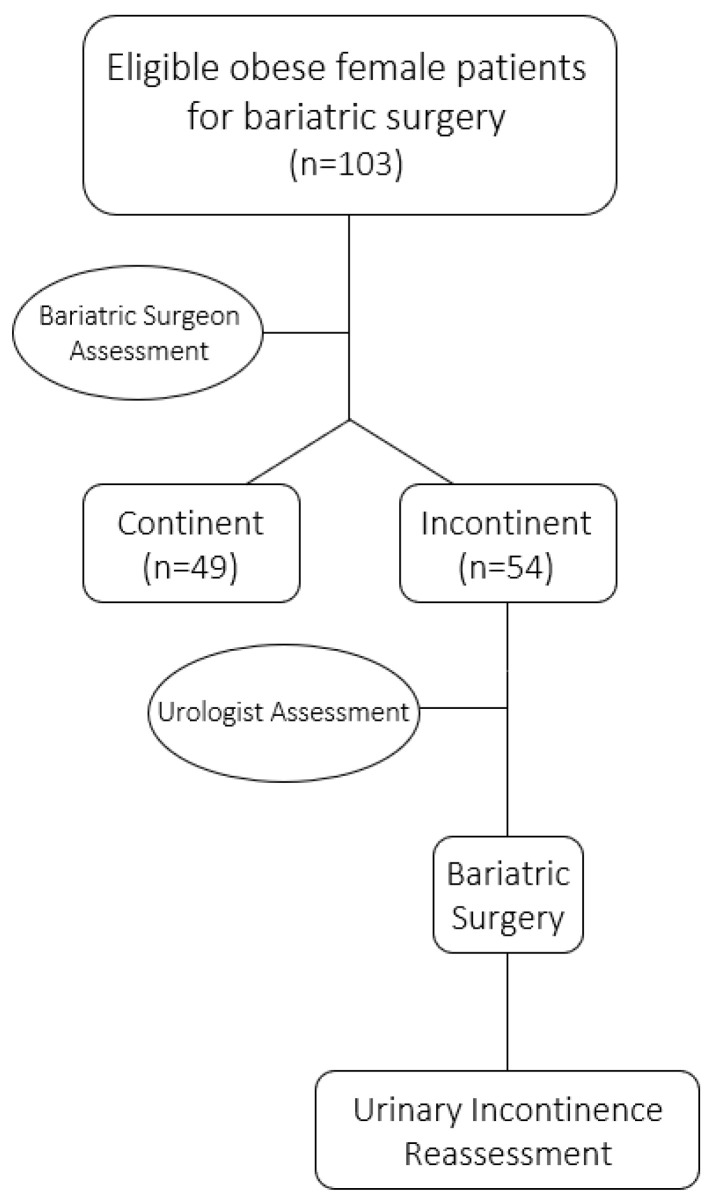
Flow diagram for study participants.

Over a period of 9 years, 54 obese female patients with urinary incontinence underwent bariatric surgery. As Figure 1 shows, over 50% of the eligible women for bariatric surgery were suffering from urinary incontinence. Table 1 describes the demographic characteristics of patients.

The mean age for the included patients was 37.1 ± 7.93. In our series, 41 (76%) women lived in an urban area, whilst 13 (24%) came from a rural area. The median number of childbirths was 1.38 ± 1.03, with 0.42 ± 0.73 vaginal deliveries and 0.96 ± 0.84 C-section deliveries. At least one comorbidity was present in 33.3% (17) of patients. A total of 18.5% (10) of our sample had high blood pressure; 13% (7) were diabetic; and 31.5% (17) had dyslipidemia. Prior to the surgery, the median BMI was 42.5 ± 3.87, and half of the women suffered from stress urinary incontinence, while 20 (37%) were found to have urge UI; 7 women (13%) had mixed UI. Table 2 describes the mean BMI and the prevalence of UI at 18 months after bariatric surgery.

After the follow-up period, we observed a significant drop in the mean BMI in all cases (42.5 ± 3.87 vs. 30.29 ± 4.22; *p* < 0.005). The prevalence of stress UI decreased after the surgery (27 (50% of all the patients) vs. 16). Both the prevalence of urge UI (20 cases vs. 15 cases after surgery) and mixed UI (7 cases vs. 5 cases) decreased, yet without statistical significance. We speculate that the lack of statistical significance might be due to the relatively small sample size.

Subjective perception of UI improvement in patients after the surgery is shown in Table 3. The ICIQ score improved from 13.31 ± 5.18 points before surgery to 8.30 ± 4.49 points after surgery (*p* < 0.0001). Before surgery, 38 patients (70%) described severe incontinence, compared to only 20 patients (37%) after surgery. A total of 16 women (31%) reported complete cure of urinary incontinence after bariatric surgery; 12 women who suffered from SUI reported no incontinence after the surgery, while only 4 patients declared no incontinence after UUI and MUI. Every patient that scored 6 or above on the VAS was declared improved after the surgery. Data from the VAS questionnaire show improvement in 46 cases (85%). It is important to mention that all patients filled in at least “5” on the VAS score, so no worsening of symptoms after surgery was found. In 15% of cases (8 patients), no improvement was found during the follow-up at 18 months. Pad usage improved from 7.04 ± 2.79 to 3.42 ± 2.77 (*p* < 0.001) per day. The number of patients using more than one pad per day decreased from 35 (65%) to 9 (17%).

We analyzed if age, initial BMI and the number or the method of delivery could predict a better improvement in symptoms of UI after surgery. No statistical correlation between the postoperative ICIQ-UI SF score, VAS questionnaire or cured incontinence rate and these parameters was found.

## 4. Discussion

In our study, female participants presented with weight-related issues. If UI was found, patients were administered the pre- and postoperative symptom questionnaires during the urological assessment. One limitation comes from the lack of a full evaluation of incontinence, including urodynamic tests before and after the surgery. The main outcomes are based on patients’ individual self-perception regarding the evolution of urinary incontinence symptoms. However, in a recent study, Okuyan et al. concluded that non-complex UI patients benefit from appropriate treatment regardless of urodynamics evaluation [27]. Obesity increases the risk of lower self-esteem and depression among patients [28], thus a bias may occur even when evaluating UI. We speculate that patients may overrate the impact of UI before surgery. Once they lose weight, their self-esteem may increase, leading to an undervalue of their UI symptoms.

UI, especially SUI, may lower a patient’s quality of life (QOL) since it affects many parts of their everyday lives, including their connections with their families, their jobs and their sexual function. People who experience UI find it difficult to accept it because of how it negatively affects their daily life, including sexuality and privacy, which can result in lower self-esteem and depression [29,30]. The burden of patients with urinary incontinence can be more severe than many life-threatening diseases. Therefore, our study mainly focuses on the patient’s perception of urinary incontinence rather than on objective parameters.

It is known that obesity is a changeable risk factor for UI [10,31]. In obese patients, central adiposity may significantly enhance pressure within the abdomen and bladder and may cause an increase in urethral mobility, leading to SUI. Another explanation implies that the pudendal nerve may become chronically stretched due to chronically increasing pressure, which may cause pelvic floor muscles to weaken. Inflammation and diabetes mellitus, which are risk factors for incontinence, are also connected to obesity [32]. In our study, 50% of women with a BMI over 33 kg/m^2^ declared a type of UI. In a combined case–cohort study, Durigon Keller and colleagues observed a 65% prevalence of UI in obese women who underwent bariatric surgery [32]. In a randomized, controlled clinical trial, Subak et al. suggested that the lower bladder pressure may be the cause of the therapeutic effect of weight loss on UI. In their clinical and urodynamic findings, a reduction in waist circumference and, therefore, pressure on the bladder were independent predictors of improved incontinence after weight loss [17]. However more studies are needed in order to explain the pathophysiology between obesity and UI.

Using data from over 14,000 patients in a study conducted by the National Center for Health Statistics in USA, Trivedi and colleagues found a higher prevalence of obesity in rural areas compared to urban areas (35.6% vs. 30.4%, *p* < 0.01). They concluded that people in rural areas were 1.19 times more likely to be obese than people in urban areas (95% C.I.: 1.06, 1.34) [33,34]. On a smaller scale, Svensson E. conducted a similar study in Sweden. The mean BMI and prevalence of obesity among women were lower in urban areas vs. rural areas [35]. Although the prevalence of obesity might be the same in urban and rural areas, in our sample, only a quarter of patients were from a rural area. This might be explained due to the precarious access of people from some rural areas to medical services.

Given the constant growth in the prevalence of obese patients, treating obesity and UI remains a very challenging task [7]. Behavioral interventions proved to be an effective method of losing weight in some obese patients. However, maintaining a long-term low weight is hard to achieve [36]. A recent meta-analysis conducted by Sheridan W. concluded that compared to behavioral therapies, bariatric surgery was linked to a considerably lower UI prevalence and maintained weight loss [37]. Nevertheless, bariatric surgery may lead to some postoperative complications that need to be considered in obesity management and discussed with the patient.

SUI and quality of life improved 18 months after bariatric surgery in our study. According to the study by Bump et al., the pathophysiological process may be linked to a drop in abdominal pressure that is to blame for involuntary loss of urine [38]. They examined the impact of bariatric surgery on urine incontinence by administering a urinary incontinence questionnaire and a urodynamic examination both before and after gastric bypass. Nine patients out of twelve had an improvement in symptoms. Although urodynamic examination was not carried out in our study, 46 patients out of 54 (85%) declared an improvement in symptoms after bariatric surgery according to the VAS questionnaire.

Prior to surgery, half of our patients were diagnosed with SUI. In this group of patients, we observed the best results in terms of symptomatic improvement. After an almost 12-point decrease in median BMI after surgery (42.5 ± 3.87 vs. 30.29 ± 4.22), 12 of 27 SUI patients declared no incontinence after surgery. UUI and MUI were also improved, but the number of patients did not carry statistical significance. A meta-analysis and systematic review from Yung Lee and colleagues assessed the effect of bariatric surgery on UI, including 33 cohort studies with over 2900 patients [39]. After bariatric surgery, resolution or improvement in any UI was found in 56% of patients. In the SUI group, 47% of patients benefitted from improvement and 39% reported a total cure of incontinence. Moreover, in their analysis, UI symptoms improved in 53% of patients from the UUI group. The ICIQ score significantly decreased by four points after surgery. Compared with our results, the data suggest a clear improvement after surgery in the SUI group and a better quality of life declared by patients regarding the type of UI.

In our sample, the ICIQ score dropped from 13.31 ± 5.18 to 8.30 ± 4.49 (*p* < 0.0001). The number of pads used daily also decreased (7.04 ± 2.79 vs. 3.42 ± 2.77; *p* < 0.0001), and only 9 patients of 54 needed more than one pad/day after the surgery. Based on ICIQ only, 20 patients declared severe incontinence after surgery. C.J.O’Boyle et. al. had similar results in a prospective cohort study with 82 female patients [40]. Over a median follow-up of 15 months after surgery, the mean ICIQ-SF score dropped by 4.4 (SD = 5.5), from 9.3 (4.4) before surgery to 4.9 (SD = 5.3) after surgery. Furthermore, the number of patients needing daily pads decreased by 48% from 65% to 17%, and one-third of patients declared no incontinence after surgery.

Pad weighing was first described as a diagnostic and assessment method for UI by James et al. in 1971 [41]. Until today, the number of pads used daily by patients with UI remains one of the best methods to evaluate the severity of UI. Although it is a non-invasive and relatively simple method, results might be subject to patients’ subjectivity. Behavioral changes, such as fluid restriction and inactivity, might undervalue the severity of UI. Furthermore, a fully continent patient may use an unnecessary pad/day after treatment of UI, considering the remaining social anxiety priorly caused by UI. Although it is as subjective as the ICIQ-SF score, the number of pads remains a useful measure for clinical care [42].

Finally, we found no statistical correlation between the ICIQ-SF score, number of pads and the severity of incontinence regarding age, BMI and both the number and method of child delivery. However, Rodrigues AFS et. al. found that age, vaginal delivery and menopause are an important risk factor for SUI persistence after bariatric surgery [43]. In the six months following bariatric surgery, menopause was the most important predictor of SUI persistence. Menopausal women were 2.7 times more likely than non-menopausal women to experience SUI persistence following surgery. They also found that each centimeter of gain in waist circumference prior to surgery increased the risk of SUI by 5.7% (*p* = 0.05). In another study, when BMI and waist circumference were combined, 2702 women between the ages of 42 and 52 showed an increased risk of SUI with every centimeter of waist circumference growth (OR = 1.04; 95%CI: 1.02–1.06), but not with unit increments of BMI (OR = 0.99; 95%CI: 0.95–1.04) [44]. Given the fact that a greater abdominal pressure caused by central adiposity might be the mechanism for SUI, waist circumference may be a better variable to take into account when assessing SUI in obese patients.

A limitation for our study comes from the relatively small sample size (54 patients) and a short follow-up period (18 mo.). Also, it was a non-randomized study, and no control group was used. We only focused on female patients considering that urinary incontinence in males is more complex and subject to more variables. Another potential limit we identified is that the initial screening for UI was conducted by a bariatric surgeon, but we consider their task to be very simple and could not induce any bias in the selection of our patients. We continue to gather data both from the already included patients who come for follow-up visits and from new patients included in our study. We consider that once the sample size grows larger, evidence for improvements in UI after bariatric surgery will become statistically significant in all subgroups defined by type of incontinence.

## 5. Conclusions

Obesity is a rapidly expanding public health problem. There are more publications examining the benefits of bariatric surgery for urinary incontinence in the literature. Half of the obese female patients eligible for bariatric surgery in our study reported symptoms of urinary incontinence. Patients who are candidates for bariatric surgery should be advised that improvement in UI may also be a significant benefit of their intervention. Our data show that bariatric surgery is able to cure urinary incontinence in one of three obese women. A significant improvement was obtained in more than two-thirds of the patients, regardless of the type of incontinence. Almost half of the patients with stress urinary incontinence declared no involuntary leak of urine after surgery. The findings of this study suggest that weight loss via bariatric surgery is an efficient method of managing SUI in obese women. A larger sample is needed to demonstrate the beneficial effect on urgency UI and mixed UI. For an obese female with urinary incontinence, treatment for obesity should prevail and incontinence should be treated only if symptoms remain after surgery.

## Figures and Tables

**Table 1:** Demographic characteristics of patients.

Variable	Value
Age, years	37.1 ± 7.93
Height, cm	159 ± 6.53
BMI, kg/m^2^	42.5 ± 3.87
Parity	1.38 ± 1.03
Vaginal deliveries	0.42 ± 0.73
C-section deliveries	0.96 ± 0.84
Only C-section deliveries	0.83 ± 0.37
Urban	41 (76%)
Rural	13 (24%)
Hypertension	10 (18.5%)
Diabetes	7 (13%)
Dyslipidemia	17 (31.5%)
Stress UI	27 (50%)
Urge UI	20 (37%)
Mixed UI	7 (13%)
Nr of pads/day	7.04 ± 2.79

**Table 2:** BMI and prevalence of UI before and after surgery. In some patients, symptoms improved significantly, although they were still present.

Variable	Before Surgery	After Surgery	*p* Value
BMI, kg/m^2^	42.5 ± 3.87	30.29 ± 4.22	0.005
Stress UI	27 (50%)	16	0.005
Urge UI	20 (37%)	15	0.163
Mixed UI	7 (13%)	5	0.3

**Table 3:** Quality of life assessments before and after surgery.

Results	Before	After	*p* Value
ICIQ mean	13.31 ± 5.18	8.30 ± 4.49	0.0001
Severe incontinence	38 (70%)	20 (37%)	0.0005
No incontinence	0	16 (31%)	n/a
VAS improvement	0	46 (85)	n/a
More than 1 pad/day	35 (65%)	9 (17%)	n/a
Nr of pads/day	7.04 ± 2.79	3.42 ± 2.77	0.0001

## Data Availability

Not applicable.
